# Relationship between skeletal Class II and Class III malocclusions with vertical skeletal pattern

**DOI:** 10.1590/2177-6709.24.4.063-072.oar

**Published:** 2019

**Authors:** Sonia Patricia Plaza, Andreina Reimpell, Jaime Silva, Diana Montoya

**Affiliations:** 1 Fundación Centro de Investigación y Estudios Odontológicos - UniCIEO, Departamento de Ortodoncia (Bogotá, Colombia).

**Keywords:** Cephalometry, Malocclusion, Regression analysis

## Abstract

**Objective:**

The purpose of this study was to establish the association between sagittal and vertical skeletal patterns and assess which cephalometric variables contribute to the possibility of developing skeletal Class II or Class III malocclusion.

**Methods:**

Cross-sectional study. The sample included pre-treatment lateral cephalogram radiographs from 548 subjects (325 female, 223 male) aged 18 to 66 years. Sagittal skeletal pattern was established by three different classification parameters (ANB angle, Wits and App-Bpp) and vertical skeletal pattern by SN-Mandibular plane angle. Cephalometric variables were measured using Dolphin software (Imaging and Management Solutions, Chatsworth, Calif, USA) by a previously calibrated operator. The statistical analysis was carried out with Chi-square test, ANOVA/Kruskal-Wallis test, and an ordinal multinomial regression model.

**Results:**

Evidence of association (*p*< 0.05) between sagittal and vertical skeletal patterns was found with a greater proportion of hyperdivergent skeletal pattern in Class II malocclusion using three parameters to assess the vertical pattern, and there was more prevalent hypodivergence in Class III malocclusion, considering ANB and App-Bpp measurements. Subjects with hyperdivergent skeletal pattern (odds ratio [OR]=1.85-3.65), maxillary prognathism (OR=2.67-24.88) and mandibular retrognathism (OR=2.57-22.65) had a significantly (*p*< 0.05) greater chance of developing skeletal Class II malocclusion. Meanwhile, subjects with maxillary retrognathism (OR=2.76-100.59) and mandibular prognathism (OR=5.92-21.50) had a significantly (*p*< 0.05) greater chance of developing skeletal Class III malocclusion.

**Conclusions:**

A relationship was found between Class II and Class III malocclusion with the vertical skeletal pattern. There is a tendency toward skeletal compensation with both vertical and sagittal malocclusions.

## RESUMO

**Objetivo:**

o objetivo do presente estudo foi estabelecer a relação entre os padrões esqueléticos sagitais e verticais, e avaliar quais variáveis esqueléticas podem influenciar na chance de desenvolver uma má oclusão de Classe II ou de Classe III esquelética.

**Métodos:**

foi feito um estudo transversal, cuja amostra incluiu radiografias cefalométricas laterais pré-tratamento de 548 pacientes (325 mulheres, 223 homens), com idades entre 18 e 66 anos. O padrão esquelético sagital foi estabelecido por meio de três parâmetros distintos de classificação (ângulo ANB, Wits, App-Bpp); e o padrão esquelético vertical, por meio do ângulo SN.Plano Mandibular. As variáveis cefalométricas foram aferidas utilizando-se o *software* Dolphin (Imaging and Management Solutions, Chatsworth, Calif, EUA) por um avaliador previamente calibrado. A análise estatística foi realizada por meio do teste qui-quadrado, teste ANOVA/Kruskal-Wallis e modelo de regressão multinomial ordinal.

**Resultados:**

foram encontradas evidências de associação significativa (*p*< 0,05) entre os padrões esqueléticos sagitais e verticais, com maior proporção do padrão esquelético hiperdivergente nas más oclusões de Classe II, segundo os três parâmetros utilizados. Na Classe III, houve maior proporção do padrão esquelético hipodivergente, considerando-se os parâmetros ANB e App-Bpp. Pacientes com padrões esqueléticos hiperdivergentes (*odds ratio* [OR] = 1,85 - 3,65), prognatismo maxilar (OR=2,67 - 24,88) e retrognatismo mandibular (OR=2,57 - 22,65) apresentaram chance significativamente maior (*p*< 0,05) de desenvolver má oclusão esquelética de Classe II. Por outro lado, pacientes com retrognatismo maxilar (OR=2,76 - 100,59) e prognatismo mandibular (OR=5,92 - 21,50) apresentaram uma chance significativamente maior (*p*< 0,05) de desenvolver má oclusão esquelética de Classe III.

**Conclusões:**

Foi encontrada uma associação entre as más oclusões de Classe II e Classe III e o padrão esquelético vertical. Há uma tendência à compensação esquelética tanto nas más oclusões verticais quanto nas sagitais.

## INTRODUCTION

According to Bjork and Skieller^1^, the total rotation of the mandible during growth is the result of the combination of matrix rotation (with its center at the condyles) and the intramatrix rotation (with its center somewhere in the corpus). These rotation patterns suffer marked variations in individuals with hyperdivergent or hypodivergent vertical patterns. Isaacson et al.^2^ stated that during growth, it is necessary to have an equilibrium between the vertical growth in the anterior face (facial sutures and/or alveolar processes) and the vertical growth at the posterior face (mandibular condyle). If the anterior face growth exceeds the posterior face growth, the mandible will rotate backward and vice versa. 

The relationship between sagittal and vertical skeletal pattern during growth have been studied by some authors^3^ ^-^ ^6^ both in longitudinal and cross-sectional studies and in different malocclusions. Riesmeijer et al.^4^ compared the craniofacial Class I and Class II growth patterns. The findings showed that the Class II samples had greater SNA and SN.GoMe angles. Chung and Wong^5^ studied the changes in craniofacial growth in skeletal Class II subjects aged from 9 to 18 years, with low, medium and high SN-mandibular plane angle; finding that, at the age of 9, the high angle group showed greater convexity, larger Y-axis and gonial angles, and greater anterior facial height. However, at the age of 18, all groups showed a decrease in facial convexity (a more flattened face) and more mandibular forward rotation. On the other hand, Mouakeh^7^ found a significantly smaller vertical face dimension and shorter lower anterior facial height (ANS-Me) in Syrian children with Class III malocclusion. Likewise, the trend toward a compensatory mechanism from the skeletal^6^ ^-^ ^10^ and dental^5^ ^,^ ^11^ ^-^ ^13^ structures when deviations occur in growth patterns is used to try to preserve a proportional and equilibrated facial pattern. 

Although the studies^1^ ^-^ ^3^ in the growth patterns of the maxilla and mandible emphasize the value of vertical growth and its relation to anteroposterior growth, vertical and sagittal malocclusion tends to be studied separately. 

To date, very little research^2^ ^,^ ^3^ has focused on the relationship between sagittal and vertical skeletal patterns. On the other hand, sagittal jaw relationships have been measured with different cephalometric parameters due to the difficulty of finding a stable reference plane. Riedel^14^ proposed ANB angle, and Jenkins^15^ used the functional occlusal plane to assess anteroposterior maxillary and mandibular relationships. Subsequently, it was popularized by Jacobson^16^ as the “Wits appraisal”, and Nanda and Merril^17^ used the palatal plane to evaluate the perpendicular projection from points A and B (App-Bpp), to represent the maxillomandibular relationship. Many authors have pointed out the flaws of these analysis: ANB angle is affected by the position of the nasion point and jaw rotations,^18^ the Wits appraisal is affected by the cant of the occlusal plane and the vertical growth of the maxilla and mandible,^17^ and the App-Bpp could be affected by the palatal plane rotation. Therefore, if a true relationship between the vertical and sagittal skeletal pattern exist, the results must be similar with the three sagittal classification parameters. To our knowledge, there are no studies that have evaluated the cephalometric variables which contribute the most to develop skeletal Class II and Class III malocclusions through the analysis of regression models. Our aims in this study were to establish the association between sagittal and vertical skeletal patterns and assess which cephalometric variables contribute to the possibility of developing a skeletal Class II or Class III malocclusion.

## MATERIAL AND METHODS

This is a cross-sectional study, based on available data from patients at the Orthodontics Department, from March 2010 to February 2016. The protocol of the study was approved by Ethics Committee of *Fundación Centro de Investigación y Estudios Odontológicos* (UniCIEO), and all patients gave written informed consent for the use of their orthodontic data for research. The research was conducted in full accordance with the World Medical Association Declaration of Helsinki. 

From the initial population of 2,186 Hispanic subjects with clinical records taken during the 6-year study period, 800 subjects who met the selection criteria were selected. By simple random sampling, 548 subjects were obtained. The sample size was calculated from the data obtained from a previous study^20^ using the OpenEpi software (Open source epidemiologic statistics for public health version 3.01, AGDean, KM Sullivan, MM Soe, Atlanta, GA, USA). A minimum of 470 subjects was required, with an estimated prevalence of Class II malocclusion of 47%, to establish an estimate within 4% of this value and a 95% conﬁdence level. The inclusion criteria were subjects with 18 years or older who had lateral cephalometric radiographs in good condition. Subjects with craniofacial anomalies or previous orthodontic/orthopedic treatment were excluded. All the radiographs were obtained following a standard protocol with Ortophos XG plus DS/Ceph (Sirona Dental Systems, Bernsheim, Germany) and an adjusted voltage of 60-77 kV, 8-15 mA and exposure time of 9.4-14.1 s. The cephalometric variables were measured using Dolphin Imaging v. 11.8 software (Dolphin Imaging and Management Solutions, Chatsworth, Calif, USA) by one previously calibrated examiner on digital lateral cephalometric radiographs on the same computer, under the same conditions of light and environment, and with rest periods to avoid fatigue. From the total samples, 50 radiographs were randomly selected, and all variables were re-measured after two weeks, to test intra-operator reliability, using Bland-Altman plots. Random and systematic errors were calculated by Dahlberg formula and a dependent *t*-test, respectively. 

Twelve (linear and angular) cephalometric measurements were traced, SNA (degrees), SNB (degrees), Pg-NB (mm), ANB (degrees), Wits (mm), App-Bpp (mm), SN.GoGn (degrees), CoA (mm), Co-Gn (mm), GoPg (mm), ArGoGn (degrees) and SN (mm). Sagittal skeletal patterns were classified according to three classification parameters (ANB angle, Wits appraisal and App-Bpp), and the vertical skeletal pattern was estimated by SN.GoGn angle, according to the values previously reported by Riedel^14^, Ghani^19^ and Nanda^17^. Additionally, for statistical purposes, the values for SNA (maxillary position) and SNB (mandibular position) were categorized. 

The pre-established cut-points for classification were:

» Anteroposterior skeletal pattern: Class I = ANB angle between 2° and 4°, Wits appraisal -3 to +3 mm, App-Bpp 3 to 7 mm; Class II = ANB > 4°, Wits appraisal > +3mm, App-Bpp > 7mm; Class III = ANB < 2°, Wits appraisal < -3mm, App-Bpp < 3mm.
» Vertical skeletal pattern: Normal: SN.GoGN= 27° - 36°; Hyperdivergent: SN.GoGN >36°; Hypodivergent: SN.GoGN <27°.
» Maxillary position: Normal: SNA = 80°-84°; Maxillary retrognathism: SNA < 80°; Maxillary prognathism: SNA > 84°.
» Mandibular position: Normal: SNB = 78°-82°; Mandibular retrognathism: SNB < 78°; Mandibular prognathism: SNB >82 °.

### Statistical analysis

The data was processed using the STATA 14 software (v. 14; StataCorp, College Station, TX, USA). The significance level was set at 5%. Applied analyses were the following: (1) Chi-square test was applied to evaluate the association between the type of sagittal and vertical skeletal patterns; (2) Analysis of variance (ANOVA) was used to establish the association between sagittal or vertical skeletal patterns with the cephalometric variables and age - when this type of analysis was not appropriate, the Kruskal-Wallis test was used and post-hoc test of Bonferroni or Sidak were done -; and (3) the analysis of ordinal multinomial logistic regression evaluated the association between the sagittal skeletal pattern (Class I, Class II, Class III) with the three classification parameters and the other cephalometric variables studied. Variables with a *p* value ≤ 0.20 were included in the multinomial logistic regression model (backward stepwise procedure). The quality of the models’ adjustments was analyzed by the log of likelihood ratio and the Akaike information criterion (AIC). The models with the lowest value of log-likelihood ratio and AIC were selected. 

## RESULTS

The random errors were within acceptable limits, varying from 0.14 to 0.33, and there were no statistically significant systematic errors (*p*> 0.10). The Bland-Altman plots indicated high intraobserver agreement, with an average error between -0.20 and 0.10 (95% CI = -0.30 - 0.70). The samples descriptive statistic is summarized in Table 1. The proportion of male and female sagittal and vertical skeletal patterns were not significantly different (*p*> 0.05); therefore, male and female cephalometric measurements were pooled.

**Table 1:** Sample´s descriptive statistics

CATEGORICAL VARIABLES				
Variable name	n		Frequency (%)	
Sex				
Female	325		59.31	
Male	223		40.69	
ANB sagittal skeletal pattern				
Class I	244		44.53	
Class II	275		50.18	
Class III	29		5.29	
Wits sagittal skeletal pattern				
Class I	302		55.11	
Class II	188		34. 31	
Class III	58		10.58	
App-Bpp sagittal skeletal pattern				
Class I	227		41.42	
Class II	176		32.12	
Class III	145		26.46	
Vertical skeletal pattern				
Normal	315		57.48	
Hyperdivergent	141		25.73	
Hypodivergent	92		16.79	
Mandibular position				
Normal	226		41.24	
Prognathism	144		26.28	
Retrognathism	178		32.48	
Maxillary position				
Normal	214		39.05	
Prognathism	252		45.99	
Retrognathism	82		14.96	
CONTINUOUS VARIABLES				
Variable name	Mean	SD	Range	
			Minimum	Maximum
Age	30.73	10.89	18	66
SNA (degrees)	83.8	3.67	72.9	97.5
SNB (degrees)	79.82	3.75	69.7	90.3
Pog-NB (mm)	0.6	1.98	-7	10.6
ANB (degrees)	3.99	2.52	-5.9	10.4
Wits	0.69	3.43	-10.4	11.1
App-Bpp	5.25	3.94	-8.5	15.6
SN.GoGN (degrees)	32.45	5.77	14.1	48.9
Co-A (mm)	84.79	5.92	70.6	102.3
Co-Gn (mm)	110.74	8.31	89	134.9
Go-Pog (mm)	71.89	5.55	58.5	89.3
ArGoGn (degrees)	123.49	6.18	99.6	147.5
SN (mm)	64.49	4.55	52.8	79.5

When evaluating the relationship between the sagittal and vertical skeletal pattern (Table 2), evidence of an association was found (*p*< 0.05) with ANB, Wits appraisal and App-Bpp. The normal vertical skeletal pattern was the greatest proportion for both Class II and Class III malocclusion groups. The Class II malocclusion group had a greater proportion of hyperdivergent than hypodivergent skeletal pattern. Meanwhile, the Class III malocclusion group established with ANB and App-Bpp had a greater proportion of hypodivergent than hyperdivergent skeletal pattern.

**Table 2:** Association between sagittal and vertical skeletal pattern

Sagittal Skeletal Pattern (SSP)	Normal (n) %	Hyperdivergent (n) %	Hypodivergent (n) %	P-Value
ANB				
Class I (n=244)	(146) 59.84	(40) 16.39	(58) 23.77	< 0.0001****
Class II (n=275)	(150) 54.55	(99) 36.00	(26) 9.45	
Class III (n=29)	(19) 65.52	(2) 6.90	(8) 27.59	
Wits				
Class I (n=302)	(183) 60.60	(63) 20.86	(56) 18.54	0.040*
Class II (n=188)	(99) 52.66	(63) 33.51	(26) 13.83	
Class III (n=58)	(33) 56.90	(15) 25.86	(10) 17.24	
App-Bpp				
Class I (n=227)	(139) 61.23	(53) 23.35	(35) 15.42	< 0.0001****
Class II (n=176)	(92) 52.27	(70) 39.77	(14) 7.95	
Class III (n=145)	(84) 57.93	(18) 12.41	(43) 29.66	

Chi^2^ test; Statistically significant at *p < 0.05; ** p < 0.01; ***p < 0.001; ****p < 0.0001.

In the association between sagittal skeletal pattern with continuous variables (Table 3), evidence against the null hypothesis was observed (*p*< 0.05), suggesting association between the sagittal skeletal pattern established with the three sagittal classification parameters (ANB, Wits and App-Bpp) and SNB, SN.GoGn, Co-Gn and GoPg measurements. Additionally, a significant (*p*< 0.05) relationship between ANB and SNA and PgNB, Wits and ArGoGn, and both App-Bpp and Wits with Co-A was found. Meanwhile, strong evidence of association was found (*p*< 0.001) between vertical skeletal pattern and continuous variables (Table 4), with SNA, SNB, PgNB, ANB, App-Bpp (mm), CoA, CoGn, GoPog, ArGoGn and SN measurements. 

**Table 3:** Association between anteroposterior skeletal pattern and continuous variables.

Sagittal skeletal pattern	Age (years)	SNA (degrees)	SNB (degrees)	PgNB (mm)	SN.GoGn (degrees)	Co-A (mm)	Co-Gn (mm)	Go-Pg (mm)	ArGoGn (degrees)	SN (mm)
	Mean (SD)	Mean (SD)	Mean (SD)	Mean (SD)	Mean (SD)	Mean (SD)	Mean (SD)	Mean (SD)	Mean (SD)	Mean (SD)
ANB										
Class I	30.54 (10.63)	83.15 (3.50)	80.72 (3.43)	0.84 (2.11)	30.95 (5.39)	84.45 (5.71)	112.11 (7.88)	72.81 (5.16)	123.00 (6.26)	64.79 (4.49)
Class II	30.66 (10.83)	84.55 (3.65)	78.59 (348)	0.35 (1.82)	34.15 (5.58)	85.15 (5.98)	108.88 (8.11)	70.76 (5.36)	123.57 (6.05)	64.61 (4.46)
Class III	32.97 (13.46)	82.25 (3.92)	83.81 (3.89)	0.84 (2.14)	28.92 (5.80)	83.56 (6.28)	116.82 (9.16)	76.49 (6.31)	122.26 (6.53)	64.78 (5.515)
P-value	0.8236 ‡	<0.0001 **** † a.c	<0.0001 **** †a.b.c	0.0116 *‡	<0.0001 **** †a.c	0.1551 ‡	0.0001 ***‡	0.0001 ***‡	0.0915 †	0.185 †
Wits										
Class I	31.09 (10.98)	83.76 (3.68)	80.07 (3.45)	0.59 (2.06)	31.96 (5.74)	84.67 (5.94)	110.82 (8.38)	72.17 (5.60)	122.91 (6.02)	64.42 (4.63)
Class II	30.06 (10.66)	83.94 (3.79)	78.46 (3.69)	0.74 (1.88)	33.39 (5.90)	85.43 (5.71)	109.60 (7.92)	70.80 (5.19)	123.97 (6.66)	64.88 (4.50)
Class III	30.98 (11.23)	83.61 (3.25)	82.87 (3.37)	0.17 (1.83)	31.93 (5.23)	83.33 (6.28)	114.02 (8.42)	73.94 (5.74)	124.92 (5.01)	63.58 (4.19)
P-value	0.5933 ‡	0.7844 †	<0.0001 **** †a.b.c	0.1365 ‡	0.0225 *†a	0.0345 *‡	0.0016 **‡	0.0006 ***‡	0.0309 *†a	0.1531 †c
App-Bpp										
Class I	30.66 (10.31)	83.64 (3.56)	79.74 (3.06)	0.67 (2.05)	32.41 (5.24)	85.03 (6.01)	110.94 (8.01)	71.99 (5.26)	123.63 (576)	64.78 (4.63)
Class II	30.44 (11.08)	83.88 (3.85)	77.61 (3.30)	0.49 (1.79)	34.68 (5.60)	85.37 (6.02)	108.92 (8.33)	70.44 (5.67)	123.96 (6.59)	64.70 (4.49)
Class III	31.17 (11.58)	83.97 (3.62)	82.59 (3.43)	0.61 (2.09)	29.80 (5.68)	83.70 (5.55)	112.63 (8.36)	73.50 (5.39)	122.67 (6.27)	63.77 (4.43)
P-value	0.8274 ‡	0.6693 †	<0.0001 **** †a.b.c	0.7192 ‡	<0.0001 **** †a.b.c	0.0435 *‡	0.0006 ***‡	0.0001 ***‡	0.1589 †	0.0879 †

Statistically significant at: *p = 0.05; **p = 0.01; ***p = 0.001; ****p = 0.0001; † ANOVA test; ‡ Kruskal-Wallis test. Post-hoc: statistical significant at:

a = Class II vs Class I; b = Class III vs Class I; c = Class II vs Class III.

**Table 4:** Association between vertical skeletal pattern and continuous variables.

	Normal (n=315)	Hyper (n=141)	Hypo (n=92)	P-value
	Mean (SD)	Mean (SD)	Mean (SD)	
Age	30.75 (10.99)	29.99 (10.21)	31.74 (11.58)	0.6360‡
SNA (degrees)	83.83 (3.48)	81.99 (2.97)	86.49 (3.63)	<0.0001**** † Δ a.b.c
SNB (degrees)	80.02 (3.13)	76.96 (2.92)	83.51 (3.29)	<0.0001**** † Δ a.b.c
Pog-NB (mm)	0.69 (1.83)	-0.35 (1.90)	1.73 (1.94)	0.0001**‡
ANB (degrees)	3.82 (2.47)	5.04 (2.27)	2.98 (2.55)	0.0001**‡
Wits (mm)	0.74 (3.39)	1.01 (3.61)	0.12 (3.30)	0.1903
App-Bpp (mm)	5.06 (3.67)	7.06 (3.85)	3.19 (3.82)	<0.0001**** † Δ a.b.c
Co-A (mm)	84.90 (5.96)	83.70 (5.66)	86.10 (5.95)	0.0114*‡
Co-Gn (mm)	110.80 (8.30)	109.57 (8.47)	112.32 (7.91)	0.0299*‡
Go-Pog (mm)	71.99 (5.50)	70.44 (5.36)	73.76 (5.42)	0.0001***‡
ArGoGn (degrees)	123.06 (5.08)	128.27 (5.10)	117.61 (5.46)	<0.0001****†Δ a.b.c
SN (mm)	64.37 (4.79)	64.30 (4.12)	65.20 (4.29)	0.2613†

Statistically significant at: *p < 0.05; **p < 0.01; ***p < 0.001; ****p < 0.0001; † ANOVA test; Δ Bonferroni; ‡ Kruskal-Wallis test; a = hyperdivergent vs. normal; b = hypodivergent vs. normal; c = hyperdivergent vs. hypodivergent.

Table 5 shows the results of the multinomial logistic regression analysis, which predicts the odds ratio (OR) of the cephalometric variables to predict the chance to have a sagittal skeletal malocclusion (Class II and Class III) and the 95% confidence interval (95% CI) for the OR. According to the results, subjects with hyperdivergent skeletal pattern [ANB (OR= 3.65, 95% CI = 1.90-6.99), App-Bpp (OR= 1.85, 95% CI = 1.10-3.12)], maxillary prognathism [ANB (OR=24.88, 95% CI = 11.31-54.70), Wits (OR=2.67, 95% CI = 1.55-4.61), App-Bpp (OR=3.80, 95% CI = 1.95-7.39)], mandibular retrognathism [ANB (OR=22.65, 95% CI = 9.42-54.43), Wits (OR=2.57, 95% CI = 1.43-4.61), App-Bpp (OR=5.42, 95% CI = 2.70-10.89)], increased CoA [ANB (OR= 1.40, 95% CI = 1.28-1.53), Wits (OR= 1.13, 95% CI = 1.06-1.21), App-Bpp (OR= 1.19, 95% CI = 1.10-1.28)], and decreased CoGn [ANB (OR= 0.78, 95% CI = 0.73-0.84), Wits (OR= 0.92, 95% CI = 0.88-0.97), App-Bpp (OR= 0.89, 95% CI = 0.84-0.94)] had significantly (*p*<0.05) greater chance of developing skeletal Class II malocclusion. The hyperdivergent skeletal pattern lost significance (*p*= 0.063) when the sagittal skeletal pattern was established with Wits appraisal. Meanwhile, subjects with maxillary retrognathism [ANB (OR= 100.59, 95% CI = 7.91-1278.84), App-Bpp (OR= 2.76, 95% CI = 1.05-7.24)], mandibular prognathism [ANB (OR= 21.50, 95% CI = 2.25-205.02), Wits (OR= 5.92, 95% CI = 2.32-15.11), App-Bpp (OR= 15.17, 95% CI = 5.90-38.98)], decreased CoA [ANB (OR= 0.58, 95% CI = 0.45-0.74), Wits (OR= 0.76, 95% CI = 0.68-0.86), App-Bpp (OR= 0.79, 95% CI = 0.72-0.86)] and increased CoGn [ANB (OR= 1.51, 95% CI = 1.26-1.82), Wits (OR= 1.19, 95% CI = 1.11-1.29), App-Bpp (OR= 1.14, 95% CI = 1.07-1.22)] had significantly (*p*< 0.05) greater chance of developing skeletal Class III malocclusion. The maxillary retrognathism lost significance (*p*= 0.927) when the sagittal skeletal pattern was established with Wits appraisal.

**Table 5:** Multinomial logistic regression model, with Class I as base.

	ANB		Wits	App-Bpp		P-value
	OR (95% CI)	P Value	OR (95% CI)	P Value	OR (95% CI)	
CLASS II						
Vertical skeletal pattern						
Normal	1		1		1	
Hyperdivergent	3.65 (1.90-6.99)	<0.0001****	1.58 (0.97-2.55)	0.063	1.85 (1.10-3.12)	0.020*
Hypodivergent	0.27 (0.13-0.56)	<0.0001****	1.15 (0.62-2.12)	0.657	0.66 (0.30-1.43)	0.292
Maxillary position						
Normal	1		1		1	
Prognathism	24.88 (11.31-54.70)	<0.0001****	2.67 (1.55-4.61)	<0.0001****	3.80 (1.95-7.39)	<0.0001****
Retrognathism	0.10 (0.41-0.24)	<0.0001****	0.79 (0.42-1.47)	0.453	0.56 (0.28-1.11)	0.097
Mandibular position						
Normal	1		1		1	
Prognathism	0.22 (0.11-0.44)	<0.0001****	0.37 (0.20-0.69)	0.002**	0.36 (0.17-0.77)	0.009**
Retrognathism	22.65 (9.42-54.43)	<0.0001****	2.57 (1.43-4.61)	0.002**	5.42 (2.70-10.89)	<0.0001****
CoA	1.40 (1.28-1.53)	<0.0001****	1.13 (1.06-1.21)	<0.0001****	1.19 (1.10-1.28)	<0.0001****
CoGn	0.78 (0.73-0.84)	<0.0001****	0.92 (0.88-0.97)	0.002**	0.89 (0.84-0.94)	<0.0001****
CLASS III						
Vertical skeletal pattern						
Normal	1		1		1	
Hyperdivergent	0.50 (0.02-1.27)	0.07	2.82 (1.15-6.91)	0.024*	0.75 (0.36-1.55)	0.442
Hypodivergent	1.67 (0.43-6.49)	0.46	0.62 (0.26-1.48)	0.281	1.60 (0.83-3.09)	0.159
Maxillary position						
Normal	1		1		1	
Prognathism	0.36 (0.09-1.41)	0.144	0.51 (0.22-1.20)	0.124	0.17(0.07-0.43)	<0.0001****
Retrognathism	100.59 (7.91-1278.84)	<0.0001****	0.94 (0.29-3.10)	0.927	2.76 (1.05-7.24)	0.039*
Mandibular position						
Normal	1		1		1	
Prognathism	21.50 (2.25-205.02)	0.008***	5.92 (2.32-15.11)	<0.0001****	15.17 (5.90-38.98)	<0.0001****
Retrognathism	0.05 (0.004-0.61)	0.020*	0.35 (0.10-1.21)	0.096	0.27 (0.10-0.71)	0.008**
CoA	0.58 (0.45-0.74)	<0.0001****	0.76 (0.68-0.86)	<0.0001****	0.79 (0.72-0.86)	<0.0001****
CoGn	1.51 (1.26-1.82)	<0.0001****	1.19 (1.11-1.29)	<0.0001****	1.14 (1.07-1.22)	<0.0001****

Statistically significant at: *p < 0.05; **p < 0.01; ***p < 0.001; ****p < 0.0001.

## DISCUSSION

Several authors^1-3^ have highlighted the importance of vertical growth, as it relates to anteroposterior growth in order to develop a proper skeletal proportion of the face. Ghafari and Macari^21^ theorized that the vertical problem can exacerbate or mask a discrepancy in the sagittal plane. Schudy^3^ promulgated that the growth of the dentofacial complex does not proceed strictly vertically and anteroposteriorly, but acting as opposing forces, each competing for the control of the pogonion, and their interplay during growth is responsible for the creation of prognathic and retrognathic facial types. The present results seem to support these theories, since strong evidence of association (*p*< 0.001) between sagittal and vertical skeletal pattern was found with all the three sagittal classification parameters used. The present study showed that the Class II malocclusion group had a greater proportion of hyperdivergent (33.51 to 39.77%) than hypodivergent (7.95 to 13.83%) skeletal pattern. Meanwhile, the Class III malocclusion group had a greater proportion of hypodivergent (27.59 to 29.66%) than hyperdivergent (6.90 to 12.41%) skeletal pattern, but only considering the ANB angle and the App-Bpp measurement. Similar to our results, Riesmeijer et al.^4^ concluded that the SN.GoMe angle was significantly greater in the Class II groups (ANB angle). On the contrary, Sidlaukas et al.^6^ found reduced vertical skeletal jaw relationship in Class II, division 1 malocclusions (dental characteristics and ANB angle). It is possible that these differences indicate ethnic background influence, but distinctive characteristics in study methods, such as the sample’s age or the severity of the skeletal pattern, are also to be considered. Spalj et al.^12^ reported that subjects with Class III malocclusion (ANB angle) and mandibular prognathism tended to exhibit a horizontal facial growth pattern. Mouakeh^7^ also showed that patients with Class III malocclusion (clinical and dental evaluation) tended to have a significantly smaller vertical facial dimension and shorter lower anterior facial height.

Having knowledge about the components of different malocclusions is of great importance in planning dentofacial orthopedic treatment in different types of skeletal patterns. Usually, clinicians tend to prioritize the sagittal problem when the correction could be more focused on the vertical problem. In Class II malocclusion with hyperdivergent skeletal pattern, therapeutic vertical control should be taken into account. Therefore, it could be relevant to include the vertical growth control in the treatment protocols, such as using high-pull headgear^22,23^ or transpalatal arch^24,25^ placed away from the palate. Furthermore, skeletal anchorage^26,27^ for molar intrusion can cause anterior mandibular rotation and thus correct both the vertical and the sagittal skeletal pattern, and improve the facial profile.

Regarding the relationship between the vertical skeletal pattern and SNA and SNB angles, the present study found statistically significant differences (*p*< 0.001) between them, with more obtuse angles in hypodivergent patterns and more acute angles in hyperdivergent patterns. Very similar results have been found by other authors^2^ ^,^ ^5^.

Additionally, the present study showed strong evidence of association (*p*< 0.001) between the anteroposterior and vertical skeletal patterns and most of the twelve cephalometric measurements evaluated. Maxillary and mandibular skeletal positions showed a compensatory trend toward both the sagittal and vertical skeletal pattern, as found by other authors^5^ ^,^ ^12^. It is important to take into consideration about these mechanisms of skeletal compensation in malocclusions when choosing the most appropriate therapy.

To our knowledge, this is the first study that has evaluated which cephalometric variables are the best predictors for the possibility of development of Class II and Class III skeletal malocclusion, evaluated with three of the most common sagittal classification parameters (ANB; Wits appraisal and App-Bpp). We found that subjects with hyperdivergent skeletal pattern (OR=1.58 to 3.65), maxillary prognathism (OR=2.67 to 24.88) and mandibular retrognathism (OR=2.57 to 22.65) had greater chance of developing Class II skeletal pattern. Meanwhile, subjects with maxillary retrognathism (OR=2.76 to 100.59) and mandibular prognathism (OR=5.92 to 21.50) had greater chance of developing Class III skeletal pattern. As for the maxillary (CoA) and mandibular length (CoGn), strong evidence of association with the anteroposterior skeletal pattern (*p*< 0.001) was shown, presenting a decrease in CoA (OR=0.58 to 0.79) and an increase in CoGn (OR=1.14 to 1.51) in Class III malocclusion, an increase in Co-A (OR=1.13 to 1.40) and a decrease in CoGn (OR=0.78 to 0.92) in Class II malocclusion. Although we could not find studies in the literature with this type of multivariate statistical analysis in order to compare our results, some authors have found similar associations between some of these cephalometric variables and Class II or Class III malocclusions. Riesmeijer et al.^4^ found statistically significantly more protrusive maxilla (SNA) and more vertical growth pattern in the Class II samples (ANB > 4°) in all age groups of both sexes. The longitudinal study developed by Jacob and Buschang^8^ using molar relationship as malocclusion classification method found a greater total mandibular length in Class I patients than in Class II, and more retrognathic mandibles in Class II than in Class I malocclusion. Sayin and Türkkahraman^28^ have also found a posteriorly positioned and rotated mandible and protrusive mandibular incisors in a sample of non-growing females. As for Class III malocclusion, despite the morphologic variability of craniofacial complex, numerous studies^7^ ^,^ ^29^ ^-^ ^31^ have reported maxillary skeletal retrusion and mandibular skeletal protrusion. In the present results, the presence of maxillary retrusion and mandibular prognathism increases the possibility of developing Class III, but with a greater OR in a retrusive maxilla. As a clinical implication of this finding, it would be advisable to use an orthopedic face mask in growing patients whose malocclusions are characterized primarily by maxillary skeletal retrusion.^32^

One of the limitations of this study was the lower frequency of subjects with Class III malocclusion (ANB = 29; Wits = 58, App-Bpp = 145), which could have affected the results in this malocclusion group. Although the prevalence of malocclusion in the anteroposterior direction was highly influenced by the sagittal classification parameter used, the majority of associations and its strength evaluated in this study remained regardless of the reference plane used for the classification of the sagittal relationship. It is important to take into account the individual biological variations before applying the results of this study to clinical practice.

## CONCLUSIONS

» Strong evidence of association (*p*< 0.001) was found between sagittal and vertical skeletal pattern, with all of the three sagittal classification parameters studied.
» According to the multinomial logistic regression model, it was observed that there is a significantly increased (*p*< 0.001) chance of developing an anteroposterior skeletal pattern in Class II if there is vertical hyperdivergent skeletal pattern, prognathism of the maxilla, mandibular retrognathic position, increased CoA, and decreased CoGn. A significant increase of chance of developing anteroposterior skeletal pattern Class III (*p*< 0.001) was noted when there is retrognathic maxilla, prognathic mandible, decreased CoA, and increased CoGn measurement.
